# A Polygenic Approach to the Study  of Polygenic Diseases

**Published:** 2012

**Authors:** D. Lvovs, O.O. Favorova, A.V. Favorov

**Affiliations:** Scientific Center of Russian Federation Research Institute for Genetics and Selection of Industrial Microorganisms “Genetika”, 1-st Dorozny proezd, 1, Moscow, Russia, 113545; N.I. Pirogov Russian National Research Medical University, Ostrovityanova Str., 1, Moscow, Russia, 117437; Russian Cardiology Research and Production Complex, 3-rd Cherepkovskaya Str., 15a, Moscow, Russia, 121552; Vavilov Institute of General Genetics, Russian Academy of Sciences, Moscow, Gubkin Str., 3, Moscow, Russia, 117809; Oncology Biostatistics and Bioinformatics, Johns Hopkins School of Medicine, 550 North Broadway, Baltimore, MD 21205, US

**Keywords:** medical genomics, pharmacogenomics, polygenic analysis, epistasis

## Abstract

Polygenic diseases are caused by the joint contribution of a number of independently acting or interacting polymorphic genes; the individual contribution of each gene may be small or even unnoticeable. The carriage of certain combinations of genes can determine the occurrence of clinically heterogeneous forms of the disease and treatment efficacy. This review describes the approaches used in a polygenic analysis of data in medical genomics, in particular, pharmacogenomics, aimed at identifying the cumulative effect of genes. This effect may result from the summation of gains of different genes or be caused by the epistatic interaction between the genes. Both cases are undoubtedly of great interest in investigating the nature of polygenic diseases. The means that allow one to discriminate between these two possibilities are discussed. The methods for searching for combinations of alleles of different genes associated with the polygenic phenotypic traits of the disease, as well as the methods for presenting and validating the results, are described and compared. An attempt is made to evaluate the applicability of the existing methods to an epistasis analysis. The results obtained by the authors using the APSampler software are described and summarized.

## INTRODUCTION

The concepts of modern genetics subdivide hereditary diseases into Mendelian and complex disorders. The Mendelian disorders are determined by carriage of a mutant variant of a single gene, whereas complex diseases depend both on a genetic component determined by the joint contribution of a large number of independent or interacting polymorphic genes and on other factors. Meanwhile, the individual contribution of each gene to the development of a polygenic disease can be small or modest. The carriage of certain allelic combinations of genes can also determine the emergence of clinically heterogeneous forms of diseases and the therapeutic efficacy of certain pharmaceutical agents.

In humans, polygenic disorders occur much more frequently than monogenic ones; they have a great social and economic impact. However, their molecular genetic nature has not been elucidated thus far. The search for the genes that are involved in the development of polygenic diseases is carried out with the use of two major strategies, namely, establishing the role of a certain candidate gene selected relying on the tentative role of its protein product in the etiopathogenesis of the disease and whole genome sequencing using the panel of genetic markers that are more or less uniformly distributed across the genome. The experimental approaches to determine the role of certain genes or the function of particular genomic regions consist in the analysis of their linkage or association with the disease.

Linkage analysis is carried out in families with several individuals affected; the role of the gene in the formation of the susceptibility to the disease can be considered to be confirmed if allelic variants that are shared between the affected individuals are revealed. Low sensitivity is a drawback of this method; therefore, methods with greater statistical power that are based on the association analysis have recently taken center stage.

An association study is an attempt to find new statistical relationships between different events or verify the already known ones. The actual causes of these relationships are often beyond the knowledge or the experimental facilities of a researcher. However, once one has collected the statistics of occurrence of combinations of different observed outcomes, a conclusion can be made regarding the significance (which is assessed based on the probability of randomly obtaining the result observed) and intensity of these relationships. The association between a certain polymorphic genome region and a phenotypic trait is analyzed by comparing the distributions of its alleles and genotypes in the representative samples of individuals, which are formed with respect to the presence/absence of this trait and need to match in terms of sex, age, and ethnicity. The allelic variants under analysis can be localized in any DNA region, including the coding sequences (exons), introns, and promoter regions of the genes, where the transcriptional regulatory regions are frequently located, as well as the other DNA regions. In exon analysis, not only the nonsynonymous substitutions determining the changes in the amino acid sequence of the protein molecule being encoded are of interest, but also the synonymous substitutions, since they can affect the mRNA structure and stability, as well as the translation kinetics due to the use of different isoacceptor tRNAs.. However, it should be remembered that in addition to the direct relation between the investigated locus and the hereditary trait, the association may be based on linkage disequilibrium between the marker locus and the true locus of the disease, if these loci are located sufficiently close to one another.

The aim of association studies is to link the phenotypic traits that are significant for medicine with such characteristics as allelic variations in the genome, epigenetic modifications, effects of environmental factors, lifestyle, etc. The phenotypic traits that are of significance for personalized medicine typically include the onset of a disease, its course (clinical presentation, extent of injury in the systems of the organism, etc.) or the efficacy of therapy with a certain drug (the area of interest of phamacogenomics). In this review, we will focus on the association between the individual traits and the carriage of allelic variants of the genome. Identification of these associations enables one to assess the risk of disease development (susceptibility), predict the character of its course, and give a preference to certain methods of prevention, diagnosis and therapy based on the features of the individual genome.

The analysis of the associations between polygenic diseases and the combined occurrence of alleles of different genes remains a relatively poorly developed research area. This can be mainly attributed to the fact that any increase in the number of genes being analyzed results in an exponential growth in the number of combinations of their allelic variants, which makes any analysis using conventional exhaustive search techniques almost infeasible. 

The present review is devoted to bioinformatic methods that search for such allelic combinations of different genes that are associated with the phenotypic traits of a polygenic disease, as well as to the methods for presenting and validating the results obtained. These methods (for the sake of brevity, they will be referred to as the polygenic analysis methods) are used to understand the cumulative effect of the genes and the nature of this effect. The association with the combination may be caused by the interplay of the phenotypic effects of the alleles on the phenotype; i.e., by nonlinear (epistatic) interaction between the genes. Alternatively, an allelic combination with a significant impact on the development of the trait can occur due to the summation of small independent subthreshold contributions of the alleles composing the combination. Both these cases will be discussed in the review.

## ASSOCIATION STUDIES

The two major types of association studies (namely, cohort studies and case–control studies) differ in terms of the time sequences in which data is collected; therefore, they also differ in terms of the parameters that can be assessed based on monitoring. In cohort studies, a selected group of individuals is divided into two subgroups; individuals who have and those who do not have a certain indicator trait (e.g., subgroups of carriers and noncarriers of a certain genotype; smoker and nonsmoker subgroups). These subgroups are monitored during a certain time interval for the development of a trait that is of interest in terms of its prediction (the target trait); e.g., a disorder. This approach enables one to numerically assess the intensity of the contribution of an indicator trait to the development of the target trait via the ratio of probabilities of disease occurrence in the carriers and noncarriers of an indicator trait. This value is assessed using the relative risk (RR).

The case–control studies are a more common type of association studies. The sample here is divided into two subgroups: the individuals who possess and those who do not possess a target trait at an instance of study (e.g., affected and healthy individuals). The presence of indicator traits that possibly affect the emergence of the disease is assessed in each group. Nothing is known about the individuals who died before the launch of the study, thus the higher the disease mortality, the less accurate the estimation of the level of association in terms of RR. The odds ratio (OR) is typically used as a criterion for the degree of difference between the carriers and noncarriers of an indicator trait in case–control studies [1]. If absolute risk of the disease in noncarriers is low, the OR and RR values are close. The higher the risk, the larger the difference between OR and RR. OR is always higher compared to RR.

The results obtained using the case–control method can be distorted because of the ethnic heterogeneity of the groups being compared or due to the environmental factors that have not been taken into account [2]. The family-based methods (e.g., comparison of the affected and healthy brothers and sisters [3]) are less susceptible to distortion. However, there are requirements for the input data (pairs of affected and healthy immediate relatives, preferably siblings, are needed) that limit their applicability for obtaining reliable dependences. The transmission disequilibrium test (TDT) [4] imposes less strict requirements on the input sample. TDT is based on the analysis of the transfer of a marker allele from heterozygous healthy parents to an affected child. The data obtained are compared with the ones expected upon Mendelian inheritance; in the case of disequilibrium of the transfer of an allele, association between the allele and the disease is inferred. The AFBAC (affected family-based control) is another family-based method of association analysis in which the control group consists of a combination of the alleles of healthy parents that have not been inherited by the affected child (one allele from each parent) [5].

In the association analysis, both the predicted (dependent) and predicting (independent) traits are the categories that divide the sample into two classes (e.g., “affected” and “healthy” or “carrier” and “noncarrier”). It is convenient to present the intersections of the classes as a 2×2 table (contingency table). Its values are used to characterize the strength of association (OR) and its significance ( *p* -value). The *p* -value is calculated using the Fisher’s exact test that was proposed in 1922 and is still widely applicable [6].

If a trait is represented by more than two classes that can be ranked (e.g., using the disease severity scale assigned by the medical community), 2 *n* -field contingency tables (where *n* is the number of gradations of a trait) are compiled; the Goodman-Kruskal gamma test is used to assess the strength and significance level of an association [7]. If ranking makes no sense, either the Freeman–Halton test that extends the Fisher’s test to more than two categories [8] or the χ ^2^ test [9] can be used.

## METHODS FOR POLYGENIC ANALYSIS

All the approaches to multivariate analysis and to polygenic association studies in particular can be divided into two fundamentally different types: 1) the use of a reduced amount of input variables based on some *a priori* data and 2) complete analysis of all available variables. The reduction of the amount of possible variables in polygenic studies involves selection of several candidate genes to carry out the association analysis [10]. This approach allows one to considerably reduce genotyping costs and the space of analysis, thus reducing its complexity and the time required for computations. On the other hand, if a gene effect manifests itself only in combination with other genes and is not observed upon its individual consideration (i.e., there is no marginal effect [11], [12]), the probability that this gene will be selected as a candidate gene is extremely low, although its role may be significant. Genome-wide association studies (GWAS) [13–16] are currently gaining popularity due to the development of both computation and genotyping technologies. GWAS belongs to the second type of polygenic analyses, i.e., the analysis of all available variables.

When analyzing genome-wide data, one inevitably encounters many extremely rare alleles. Individual consideration of these alleles does not allow one to arrive at a conclusion regarding the impact of each allele on the disease. However, when considering the effect of several alleles altogether, the observed data can be sufficient to validate the assumption that they have a combined effect. In other words, data on each of the rare alleles is insufficient; however, that data should not be neglected, since association can be reliably established when data on several rare alleles is accumulated. This effect is known as the additive effect; it can also be observed for objects other than rare alleles. However, in the case of rare alleles, additive effect detection is one of the most promising methods for an association study. Correspondingly, the theory attributing the emergence of a large number of common diseases to the carriage of rare alleles is named CDRV (common disease / rare variant). This theory, which is currently gaining common acceptance, is an alternative to the CDCV (common disease / common variant) theory. A set of methods have been specially designed for the assessment of the additive contribution of rare alleles, e.g., the combined multivariate and collapsing (CMC) method [19], weighted sum statistics [20], and the gene burden test [21].

The problem of correcting for multiple hypothesis testing becomes especially urgent upon polygenic analysis. This problem can be briefly formulated in the following way: an increasing number of tested hypotheses results in an increase in the probability of a random (including unlikely) outcome, which reduces the significance of the postulate that the statistical relationships observed represent specific non-random dependences.

**Fig. 1:**
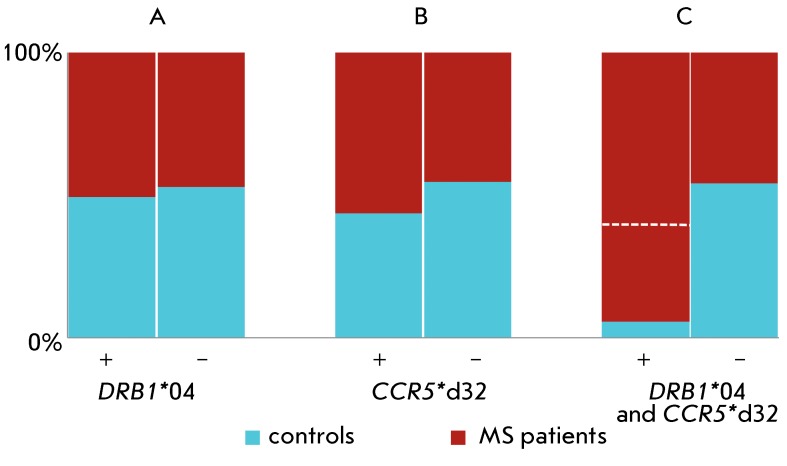
Visualization of 2x2 tables of carriage by the MS patients and control group individuals of: alleles of the major histocompatibility complex HLA- *DRB1* (A), the chemokine receptor *CCR5* (B), and their combination (C) (based on data from [28] for ethnic Russians). Red areas correspond to the case; blue – to the control group. The ratio of the vertical fields reflects the distribution of carriers (+) and noncarriers (-) of *DRB1* *04 (A), *CCR5* *d32 (B) and a combination of *DRB1* *04 and *CCR5* *d32 (C). The horizontal dashed line in (C) corresponds to the expected ratio of the number of patients and controls among the carriers of the allele combination calculated under the assumption that the allele effects are independent.

If a number of comparisons used for studying the association of a phenotypic trait with several alleles of one highly polymorphic gene or upon simultaneous assessment of the role of several biallelic candidate genes is small (although not equal to 1), such an increase in significance is taken into account using the Bonferroni correction [22], which simply multiplies the corresponding *p-* values by the number of tests carried out. However, the Bonferroni correction turns out to be too conservative because of the underlying assumption that the tests are independent. A more accurate correction can be obtained using the Westfall–Young method [23], which does not imply independency and compares the best observation with the best results for the permuted samples. Another approach to this problem consists in assessing the false discovery rate (FDR) instead of the family-wise error rate (FWER) [24, 25].

Gene-gene interaction (epistasis) has recently turned into a widely discussed theme. This interest is to a significant extent due to the poor reproducibility of the results in assessment of the role of individual genes in the formation of susceptibility to polygenic diseases; in particular, in GWAS. There is a certain ambiguity in the terms “epistasis” and “epistatic interaction.” They were originally used to denote complete masking of the effect of a polymorphism in one locus by the polymorphism of another locus; later, it was extended to refer to any other type of influence that certain polymorphisms have on the manifestation of other polymorphisms in the phenotype. The differences in interpretations of the term “epistasis,” as well as the problems arising due to these discrepancies, have been thoroughly described in [26, 27].

The results of an analysis of the contribution of carriage of the HLA class II allele *DRB1* *04 ( *A* ), an allele with a 32 nucleotide deletion in the chemokine receptor gene *CCR5 * ( CCR5* d32) ( *B* ), and their combination ( *C* ) to the development of multiple sclerosis (MS), a typical polygenic disease, is shown in *Fig. 1* in the form of visualized 2x2 contingency tables (the experimental data were taken from [28]). In all the cases, MS patients and healthy individuals were divided into two classes based on carriage/noncarriage of the allele (homo- and heterozygotes with respect to this allele were not distinguished). The polymorphism of the *CCR5* gene was indeed biallelic (the deletion allele and the wild-type allele), whereas 18 groups of alleles of the *DRB1 * gene were analyzed for this highly polymorphic gene. The group of noncarriersof the *DRB1* *04 allele was made up of the carriers of the remaining alleles of this gene. It is clear from *Fig. 1C* that the carriage of the combination of DRB1* 04and ССR5* d32 is associated with the disease to a higher extent than might be expected based on the additive contribution of the constituting alleles. This fact can be construed as resulting from the epistatic interaction between the genes under consideration. This example is an illustration of the simplest type of polygenic analyses, when only the joint contribution of two alleles to phenotype formation is taken into account.

We have proposed the use of the odds ratios ratio (ORR) as a numeric measure of epistasis [29]. It is based on the concept that if at least two alleles within a combination do not interact with each other, the OR value for carriers of this combination will be made up of the product of the ORs of individual alleles within the combination. The product is regarded as the expected OR and compared with the observed OR. The more this ratio differs from unity, the stronger the predicted epistatic interaction between the genes.

The ORR value [29] can be used to analyze the interaction between two or more alleles. However, the absence of a method to assess the confidence interval (CI) is a significant drawback here. The Synergy Factor (SF), a measure of epistasis described in [11], has contrasting advantages and drawbacks. The method for CI calculation has been designed for it; however, this value can be used for the analysis of the interaction between two alleles (or other binary indicator traits). Both values are the ratios between the OR observed for the allelic combination and the product of the OR observed individually for its components; however, the OR values are calculated using different methods. ORR compares the number of carriers and noncarriers of the indicator trait (whether this is an allelic combination or an individual allele) in patients and the control group, as is shown in *Fig. 1* . In the case of SF, the carriers of an allelic pair are compared with the carriers of neither allele, as well as the carriers of each allele constituting the combination that are also noncarriers of another allele. Identically to the situation with ORR, SF > 1 attests to a positive (mutually enhancing) interaction, whereas SF < 1 attests to a negative (compensatory) interaction. The SF value can actually be determined for more than two alleles; however, the result will depend on their order of combination to form complex traits. Thus, it is reasonable to use both of these assessments.

The available tools for an analysis of the cumulative effect of several genetic variables use various algorithms for data mining and are discussed below.

The conventional logistic regression, in which the coefficients of model terms at the second order and higher correspond to the interaction, is the most popular method [30]. Iterative simulation is required to use this method to search for the most closely interacting allelic combination, which weakens the statistical power of the method. The two-step variant implemented in GenABEL [31, 32] allows one to solve the problem of iterative testing by using the data on dispersion in individual loci to select the ones with a higher interaction probability. Various heuristic approaches, such as genetic programming [33], neural networks [34], pattern mining [35], dimensionality reduction techniques [36], and Markov Chain Monte Carlo (MCMC) methods (which include APSampler [37, 38], BEAM [39, 40], and logic regression LogicReg [41–43]) are used.

The association between carriage of any combination of alleles (or another indicator trait) with the phenotype can be assessed in the same manner as is done for one allele (trait). In other words, each combination can be regarded as a compound trait and can be characterized by the significance level of association and the RR or OR values. Numerous combinations are possible; therefore, the task of searching for the combinations characterized by the most significant association moves to the fore.

The multifactor association analysis can also be carried out using family-based data. There are multiallele and multilocus versions of TDT [4] (the method that is based on McNemar’s test and was originally designed for biallelic single loci). Methods extending TDT to several allelic variants have been proposed by a number of authors. These methods include calculating the marginal homogeneity [44]; iterative grouping of alleles into two groups: the “allele under study” and “the remaining alleles,” followed by the McNemar’s test [45] and multiple testing correction; and calculation of the disequilibrium in the allele transfer using logistic regression [46], which is best suited for highly polymorphic loci. When carrying out the analysis simultaneously at several loci, methods involving the comparison of the actual child’s genotype with all the theoretical genotypes that are possible for his parents are used [45, 47, 48]. Linkage disequilibrium between the loci under analysis is either calculated from the sample or taken from known data (e.g., from HAPMAP [49] in the FAMHAP [48, 50] software).

Some commonly used tools for polygenic association analysis are thoroughly discussed and compared below.

## PLINK

PLINK freeware was developed at Harvard University [30, 51]; it is a large interrelated collection of various algorithms for the analysis of genotypic and phenotypic data, including the methods for polygenic analysis. PLINK has been used in a number of studies focused on genetic interaction (e.g., [52–55]).

One of the methods for the analysis of gene interaction in PLINK is based on the consideration of regression models [56]. The logistic regression model assuming that the probability of an event (in our case, disease) is described as a logistic function of a linear combination of independent variables (predictors) is used upon a binary outcome (e.g., “healthy–affected”) [57]. The common linear regression of the same predictors is used for quantitative phenotypes (such as three degrees of arterial hypertension). In this case, independent variables are indicator functions that can assume either a 1 or 0 value, depending on whether a certain allele or genotype is present in the genome (or upon the presence of any other indicator trait). The analysis yields a set of regression coefficients for the indicator functions of the alleles and their combinations, and the levels of the statistical significance of the values by which these coefficients differ from zero. High significance of the difference of the coefficient corresponding to a certain combination of alleles from zero attests to their association. That is how the “PLINK -epistasis” test proceeds.

The “PLINK -case-only” is a simpler test on interaction; it verifies the correlation between carriage of several genotypes by patients. If the correlation between a genotype pair is high and their linkage can be excluded from consideration, it means that they interact. This test is based on an a priori assumption that the revealed correlation is typical only of the affected individuals. The two-step procedure verifying the presence of the correlation in the total sample does not include this assumption; however, the results provided by it may still be biased [58]. The key advantages of the PLINK software include its applicability for GWAS and a wide set of analysis tools, whereas its drawback consists in the limitations on the data format, since only biallelic markers can be used for work using this software.

## MDR 

The multifactor dimensionality reduction (MDR) algorithm has been widely used for mining polygenic associations in case-control studies [59–62].

At the first step, all data is randomly divided into two sets: the training set (e.g., 9/10 of the data) and the testing set (e.g., 1/10 of the data). A parameter characterizing the ratio between the number of affected and healthy individuals carrying the combination of alleles and genotypes is determined for each combination. The combinations are classified into categories (e.g., low-risk and high-risk combinations) based on the value of this parameter. Thus, a transition is made from the *n* -dimensional space of all single polymorphic loci and phenotypes to a two-dimensional space, where the risk level is one dimension, and the carriage of a certain allelic combination is another dimension. Among all combinations, there will be one having the lowest classification error in the training (Training Accuracy) and testing (Testing Accuracy) sets. The division into groups is repeated 10 times with the parameters of the random number generator varied. The cross-validation consistency is defined as the number of cross-validation replicates out of 10 in which that same model was chosen as the best model. The model is considered to be valid if its cross-validation consistency is at least 9/10.

In addition to a text representation of the results, the MDR software package includes dendrograms showing a pairwise interaction analysis, where the type of interlocus interaction is shown with different colors (from epistasis to independence); the bond length shows the interaction strength.

## MCMC-BASED METHODS

The exhaustive search of all combinations (e.g., that used in MDR by default) loses its efficiency when the number of alleles under analysis increases because of the large number of possible combinations. The so-called combinatorial explosion occurs. Moreover, the statistical significance of the combinations obtained using this procedure becomes less obvious due to the multiple testing problem. On the other hand, simple gradient (“greedy”) methods, which refine the intermediate result in a stepwise manner, frequently yield no adequate results at all, since they are prone to trapping in local optima rather than reaching the global ones.

There are different heuristic methods enabling one to mine the global optimum without using the exhaustive search procedure. The Markov Chain Monte Carlo (MCMC) algorithm is one such method [38, 40, 63–65].

The main idea in this method is that, as with the gradient search, it strives for a better solution than the already existing one. However, unlike the gradient search, it can also proceed to a worse solution with some probability; this probability decreases as the fit to the data for the proposed solution becomes poorer.

**BEAM**

In the search for associations, the BEAM (Bayesian Epistasis Association Mapping) algorithm [40, 66] is based on the fact that the distribution of genotypes in patients with disease-associated loci differs from that in the control group. The algorithm is aimed at classifying all the loci into loci that are not associated with the disease, loci individually associated with the disease, and the associated and epistatically interacting loci. The software uses the MCMC method to find the partition of the loci set into these three classes, which is the most probable one for the given genotypes and disease degrees. The loci are regarded as epistatically interacting if the joint distribution of their alleles/genotypes fits the data better than the distribution derived from the independent model (product of the allelic/genotypic distributions). BEAM can account for haplotype data in order to differentiate them from epistatic interactions.

**Logic Regression**

The Logic Regression algorithm uses MCMC to optimize the models of regression search for polygenic associations [43, 65]. The name of the method refers to the well-known logistic regression that solves a similar problem in a different way. The indicator functions of logic combinations (logic functions) of the presence of different alleles are used as predictors of logic regression; the combination of the optimal functions is determined using MCMC. The logic functions obtained show the type of allelic interaction.

**APSampler**

The logic of the analysis of polygenic data using the APSampler software [38] differs considerably from the previously described software packages, where the predicted phenotypic trait can possess only two values (e.g., “affected” and “healthy”). The use of the nonparametric Wilcoxon test in APSampler software permits the analysis of data with more than two values of the target trait if ranking of the outcome is possible, allowing the use of a number of internationally recognized scales to define the groups for analysis. For example, in the case of stroke, such scales could be the degree of depression of consciousness, the initial severity of the disease, stroke outcome, which all have their own values and the number of values of at least three levels. The genetic pattern (i.e., the combinations of alleles and genotypes of different loci associated with a phenotypic trait) is the major object in the APSampler software for predicting an indicator trait. The pattern search is carried out using MCMC; several patterns being considered at each step simultaneously. The set of patterns is optimized from step to step in terms of the probability of all the patterns within the set being independently and simultaneously associated with a trait. The nonparametric Wilcoxon test is used to assess the probability of association of each pattern; the subsets being compared differ in carriage of only one pattern within the set. The algorithm includes two steps. The first step yields a list of patterns that have been encountered during the MCMC search and validates the findings by determining the significance of association for each pattern from the list using the Fisher’s test (in the case of a dichotomous outcome) or the Goodman’s and Kruskal’s test [7], if there are more than two categories. At the second step, the software then repeatedly mixes the labels of the phenotypic trait and runs the search for associated patterns again. The reliabilities of association based on the results of these permuted runs provide the distribution of the reliabilities of the findings on the assumption of the null hypothesis of no association. This null distribution is used to validate the combinations obtained in the first step.

The *Table* summarizes data pertaining to the functional possibilities of the described software for polygenic association analysis. The data presented in the *Table* attest to the fact that the software proposed for polygenic analysis have considerably different functions. The software being compared can be used in different instances, depending on the available genetic and phenotypic data, on the content and format of the desired results, as well as the ability of a user to run the software at the level of the command line. One also needs to make allowance for the fact that the target result notably differs for different programs. 

MDR is very convenient due to the presence of a user interface and graphical visualization of the results, including epistasis. It provides the obtained phenotype-associated loci and their combinations, whereas APSampler takes into account the direction of association, which is determined by carriage of alleles of the loci and their combinations. Both APSampler and MDR operate with polyvariant input traits, whereas the rest of the programs operate only with binary indicator traits. These two algorithms are also similar in the fact that they allow one to analyze epistatic interaction after association has been determined, whereas BEAM a priori divides all alleles into three groups: the ones with the marginal effect, the ones with epistasis, and those with no effects. The characteristics of combinations of loci, which are given by MDR, are statistically reasonable. However, their correlation with the association strength is not obvious. LogicReg provides no conventionally interpretable association values at all. APSampler and BEAM solve this problem by performing the Fisher’s exact test for the association between the resulting indicator traits and the phenotype. In general, BEAM, PLINK, MDR, and LogicReg can be applied in basic research, including in studies devoted to gene interaction or for operation within a larger integrated software environment. However, they a priori do not have the necessary set of functions to solve such applied medical and genetic tasks as searching for the markers of susceptibility or searching for pharmacogenetic markers, for which the APSampler software can be used.

**Fig. 2:**
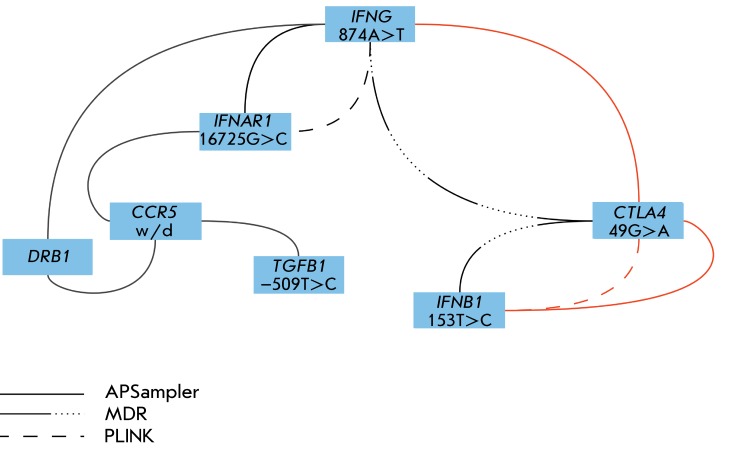
Search for biallelic combinations of immune response genes associated with the response to treatment of MS with glatiramer acetate (based on data from [29] for the ethnic Russians) with APSampler, MDR, and PLINK. APSampler [38] finds all biallelic markers found by the other software as well as identifies additional combinations. The red color marks the findings that have been validated by permutations in APSampler ( *p* < 0.1) or MDR cross-validation (CVC > 8/10).

These five programs were used with the data taken from [29] in the user mode (i.e., with all default settings). BEAM found no associations with *p < * 0.05; LogicReg required additional data processing. The results of using APSampler, MDR, and PLINK are given in *Fig. 2* . It is clear that APSampler found both the combinations found by MDR and those found by PLINK; moreover, all the validated findings of APSampler have also been validated by at least one of these programs.

## STUDIES PERFORMED USING APSAMPLER

A large number of studies using the APSampler software have been carried out since the first publication [38]; the authors participated in most studies due to the fact that at the initial stages of development, the software was relatively difficult to operate. This fact allowed them to upgrade the software according to the users’ requests, supplement it with new features broadening the potential of validation [67], data management, visualization of the results and the help files elucidating the use and structure of the APSampler software. At the moment of writing, the software is open-source and can be used free of charge [37].

The authors used the APSampler software to analyze the cumulative effect of the alleles of a number of candidate genes on the development of multiple sclerosis (MS) [68], different forms of arterial hypertension [69–71], myocardial infarction [72], ischemic stroke (IS) [73, 74], and hemorrhagic stroke [75]. The studies were carried out in compliance with the principle of ethnic homogeneity in Russians or Yakuts. The Yakut population is of particular interest in terms of ethnogenomics, since the founder effect and a certain geographic and cultural isolation are observed in it [76]. APSampler was also used in pharmacogenetic studies of MS for the investigation of the relationship between the genetic status in patients and the efficacy of treatment with immunomodulatory drugs, interferon beta (in Irish patients, [67]) and glatiramer acetate (in Russian patients, [29, 77]).

**Table 1:** Brief comparison of the potential of different software for polygenic association analysis

	APSampler [38]	BEAM [40]	LogicReg [43]	MDR [60]	PLINK [30]
Graphical user interface	-	-^1^	-^2^	+	+
Binary phenotype	+	+	+	+	+
Quantitative rank phenotype	+	-^3^	-	-	+
Working with missing data	+	+	+	-^4^	+
Statistical mining of combinations of particular alleles associated with phenotype	+	+	+	-^5^	+^6^
Assessment of the association for the established combinations using the Fisher’s exact test	+	+	-	-	-
Validation procedure	+	+	+	+	-
Polyallelic loci	+	-^7^	-	+	-
Mining epistasis	+^8^	+	+	+	+
Graphical representation of epistasis	-^9^	-	-	+	-
Possibility of carrying out the association analysis for the allelic combination specified by the user	+	-	-	+	-^10^
Genome-wide analysis	-	+	-	-^11^	+
Possibility of using the command line to run software (e.g., on a server).	+	+	+	+	+
Available for UNIX	+	+	+	+	+
Available for Windows	+	+	+	+	+
Parallel computing	+	-^12^	-	-^11^	-

There is a version of the BEAM software integrated into the GALAXY server application [83].

The algorithm has been used in the software environment for statistical computing and graphics R [84].

The software automatically divides the data into two categories using the mean value.

The authors propose specialized software, MDR Data Tool [85], for filling in the missing values.

The software finds the interacting and phenotype-associated loci rather than their alleles.

Only pairwise mining is available.

The number of alleles in each locus has to be equal.

Despite the fact that mining of epistatically interacting alleles has not been claimed to be a specific function of the APSampler software, the experience of practical use of the software attests to the possibility of using it for mining epistasis.

Perl software for graphical representation of epistasis has been designed [37].

The haplotype-association analysis is proposed.

A specialized software has been provided for this purpose [86].

Specialized software PBEAM for parallel computing [87].

In most of the aforementioned studies, the group of nonrelative patients was compared pairwise with the control group of unaffected nonrelative individuals, which was similar to the affected sample in terms of their ethnicity, sex ratio, and the mean age. Two groups of patients with clinically heterogeneous forms of the same diseases (e.g., arterial hypertension with and without hyperaldosteronism [69]) were compared in some cases. When studying the genetic susceptibility to arterial hypertension preceding the development of IS, the patients were at first divided into two subgroups in accordance with the hypertension level. The 2×4 contingency table was subsequently used to find such allelic combinations among identified ones carriage of which is characterized by monotonous increase from normotonics to third degree hypertensive patients [71]. In pharmacogenetic studies, the patients responding and not responding to treatment were also compared pairwise using the “comparison of extremes” approach.

**Fig. 3:**
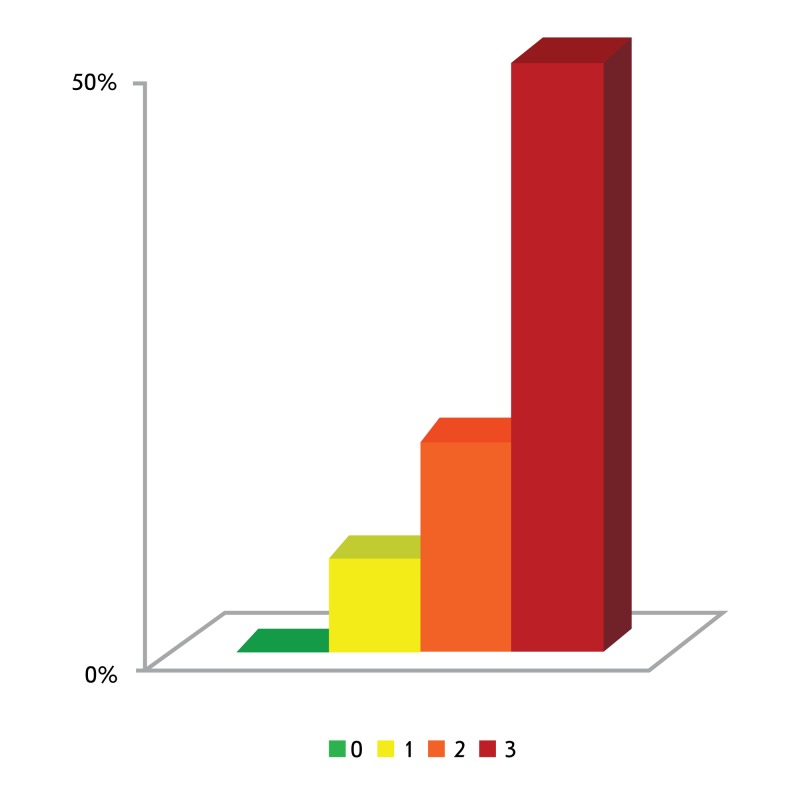
Carriage of the triallelic combination FGB* –249C + APOE* ε4 + *CMA* *–1903A, which was found with APSampler in Yakut ischemic stroke patients with different levels of the preceding blood pressure [71]. 0 – normotonics, 1–3 – first, second and third degree hypertensive patients, respectively, according to the criteria of 2003 ESH/ESC [82]. Carriage is represented as a percentage of the total number of patients in the subgroup.

The candidate genes were selected based on the existing conceptions of participation of their protein products in the processes involved in the disease pathogenesis. When analyzing the genetic susceptibility to cardiovascular diseases, the following genes were selected: the ones whose protein products participate in inflammation, the genes of hemostasis, transport, and lipid metabolism systems, the genes of the renin-angiotensin-aldosterone system, and some other genes. For MS, the candidate gene products participate in the development of the immune response and chronic inflammatory process. Polymorphic regions (mostly, single nucleotide polymorphisms, or SNPs, being of interest in terms of their function; i.e., knowingly affecting the amount or property of the encoded protein product) were usually typed in these genes. The joint contributions of 10 or more polymorphic markers have been analyzed in relatively small samples consisting of no more than 500 individuals. Although this sample size, which is typical of Russian studies, cannot be compared to the size of the groups formed by the international consortiums, we have found highly significant associations between allelic/genotype combinations and the phenotype under study using the APSampler software. This can be illustrated by the data pertaining to an association between the triallelic combination FGB* –249C + APOE* ε4 + *CMA* *–1903A and the level of arterial hypertension preceding the development of IS in the Yakut population ( *Fig. 3* ). A monotonous rise in the carriage frequency of the named triallelic combination was observed in a sample consisting of 115 patients: from 0 in normotonics to 47% of the total number of individuals in the subgroup of third degree hypertensive patients; the *p* -value assessed based on the Fisher’s test in the 2x4 contingency table was 0.0003. In this case, a vivid example of the effect of the joint contribution of the genes encoding components of three different key systems of the homeostasis, namely, hemostasis system ( *FGB* ), the lipid metabolism system ( *APOE* ), and the renin-angiotensin-aldosterone system ( *CMA* ), to the development of arterial hypertension has been observed. Thus, the disease is most likely to emerge with the summation of the independent contributions of individual genes.

The reason for such a high information value upon a rather modest amount of experimental data can be attributed to the advantages provided by the ethnic and clinical homogeneity of the groups used in the analysis, whereas groups consisting of tens of thousands of patients from different countries and patient care institutions, which are formed within the framework of consortiums, usually fulfil the homogeneity requirements in terms of neither ethnicity nor clinical presentation. This fact may smooth their genetic deviations from the control group. However, the major reason for the high information value of the results obtained using the APSampler software can presumably be attributed to the high statistical power of the analysis. Without going into the details of what underlies this phenomenon, one can summarize by saying that identification of the association of the alleles/genotypes of individual genes during the analysis of any studied disease was a rare event, whereas phenotype-associated combinations of two-four alleles were found in almost all cases. It is appropriate to note here that both positive and negative associations could be observed; a differently directed effect of the alternative alleles has been successfully revealed for most, but not all, cases.

The association of MS with the DRB1* 15 allele of the major histocompatibility complex [78, 79], with the microsatellite marker TNFa9 [80] and with the biallelic combination of *DRB1* *04 and  *CCR5* *d32 [28] (see *Fig. 1* ) in the Russian population was previously demonstrated without the use of the APSampler algorithm and reproduced in an independent sample using the APSampler algorithm [68]. The replication of the data pertaining to the association of these genetic factors with the development of MS complies with the criteria widely accepted across the world’s scientific community for validation of the results and attests to the software’s efficiency.

Based on the aforementioned observations, the concept of the minimal set (combination) of alleles as a genetic risk factor that is revealed in a certain study has been formulated [68]. This means that any subset of this set is characterized by a lower significance of association. Thus, we have identified [68] two MS-associated triallelic combinations comprising alleles of the polymorphic regions of the * DRB1* , *TGFB1, CTLA4,* and *TNF* genes. The differences between the groups of affected and healthy individuals in the carriage frequencies of the biallelic combinations and of the individual alleles within the triallelic combinations 1 and 2 did not reach the significance level ( *p*  < 0.01). It is important to note that the subgroups of individuals carrying the MS-predisposing combinations 1 and 2 did not overlap and corresponded to approximately 5 and 9% of MS patients, respectively, whereas they were not present in the control group. Thus, identically to the case of classical monogenic dominant disorders, all the carriers of either combination in our sample turned out to be affected. Identical results were obtained in our other studies. In either case, the minimum set of alleles is a compound genetic marker of the polygenic disease or of another phenotype.

**Fig. 4:**
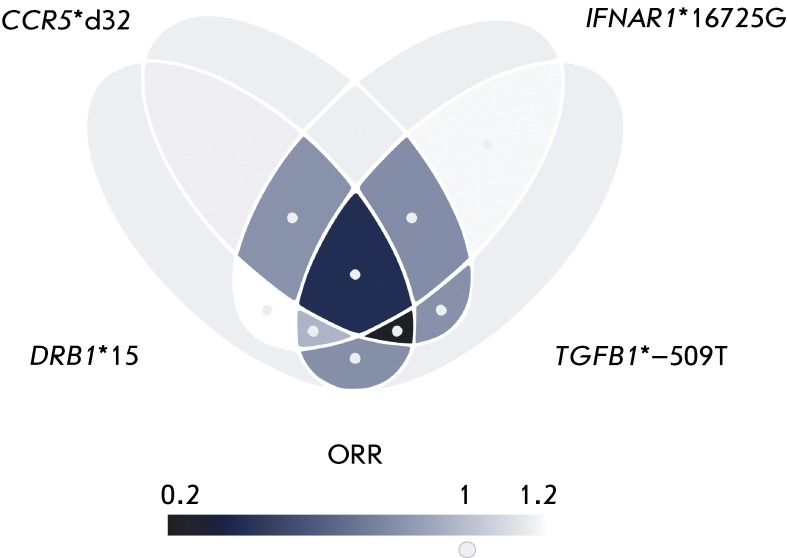
Venn diagram describing the possible interaction between the components of the *DRB1* *15 + *TGFB1* *–509T + *CCR5* *d + *IFNAR1* *16725G combination, which is negatively associated with the efficiency of the treatment of MS with glatiramer acetate, as identified using the APSampler software [29]. Each of the four ellipses in the diagram corresponds to one of the four alleles in this combination. The intersections of the ellipses correspond to all possible combinations of the four alleles, color intensity reflects the ratio of the observed OR to the expected OR (ORR), in accordance with the gradient scale provided below. The gray areas corresponding to individual alleles, as well as the small circles, correspond to the reference ORR, which is equal to 1. The more the color of an area differs from gray, the stronger the epistatic interaction of the alleles represented by the area. The values of the expected OR are calculated for each combination as a product of the ORs of the individual alleles corresponding to the overlapping areas.

We attempted to solve the question pertaining to the type of interaction between the alleles within the gene combination (epistatic or additive) in a pharmocogenetic study where the association between the efficacy of treatment of MS patients with the immodulatory drug glatiramer acetate and the allelic polymorphisms in a number of the immune response genes was analyzed [29]. The carriage of allelic combinations of four genes ( *DRB1* *15 + *TGFB1* *–509T + *CCR5* *d + *IFNAR1* *16725G) exhibited a 14-fold increase in the risk of ineffective response to glatiramer acetate therapy (OR = 0.072 [CI = 0.02–0.^28^]; *р* = 0.00018); the association withstood permutation testing ( *р*_perm_ = 0.0056), which had been included into the software by the time the study was conducted. The triallelic combination ( DRB1* 15 + CCR5* d + *TGFB1* *–509T) differed negligibly from the tetra-allelic combination as a marker of treatment inefficacy, whereas the association between all the other components of the tetra-allelic combination and treatment inefficacy was considerably weaker. Graphical visualization (the Venn diagram) of the character of the interaction between different components of the “unfavorable” allelic combination ( *DRB1* *15 + *TGFB1* *–509T + *CCR5* *d + *IFNAR1* *16725G) is given in *Fig. 4* . For the triallelic combination ( *DRB1* *15 + *TGFB1* *–509T + *CCR5* *d) ORR was 0.2 (i.e. it was fivefold lower than 1) and remained unchanged after the addition of the *IFNAR1* *16725G allele. We regard these data as evidence of the epistatic interaction between the alleles of the *DRB1* , *CCR5,* and *TGFB1* genes.

Unexpected data on epistatic interactions upon formation of genetic susceptibility to IS in the Russian population were obtained in [73]. The analysis using the APSampler algorithm has revealed the protective biallelic combinations ( *IL6* *–174C/C + *FGA* *4266A) and ( *IL6* *–174C/C + *FGB* *–249С), which were associated with IS slightly more significantly than the protective genotype *IL6* *−174C/C by itself and had practically the same OR value (0.32–0.35). Each of the alleles within these combinations ( *FGA* *4266A or *FGB* *–249С) upon joint carriage of the *IL6* G allele, which is the alternative to the *IL6* *–174C/C genotype, “neutralized” its significance as a risk allele by reducing both the significance levels and the OR values (from 2.9 to 1.9–2.1). In other words, association between IS and combinations of the alleles/genotypes of *IL6* , * FGA * and *FGB* has been observed; *IL6* played a key role, whereas the *FGA* and *FGB* genes had a modulating function. This observation presumably attests to the fact that the *FGA* and *FGB* genes contain interleukin-6-sensitive elements, which are capable of binding to STAT3 (the major transcription factor transmitting signals from the interleukin-6 receptor to the nucleus) [81]. 

## CONCLUSIONS

Searching for polygenic combinations associated with a phenotypic trait (i.e., composite genetic markers) is an adequate analysis tool for studying polygenic diseases. The statistical methods enabling this type of analysis are currently rapidly being developed.

In accordance with all the aforementioned facts, composite genetic markers can result from epistatic interaction between components or be of additive nature. Taking into account the complexity of various cumulative effects and their direction, one can claim that identification of a reliable composite marker (even if it carries a small number of components) is an important step in understanding the etiopathogenesis of the disease. Indeed, such a marker may attest to the key link in a complex regulatory network of interactions between biological macromolecules. 

## Abbreviations

CDCV: common disease / common variant
CDRV: common disease / rare variant
CI: confidence interval
CMC: combined multivariate and collapsing
FDR: false discovery rate
GWAS: genome-wide association study
MCMC: Markov Chain Monte Carlo
MDR: multifactor dimensionality reduction
MS: multiple sclerosis
IS: ischemic stroke
OR: odds ratio
ORR: odds ratios ratio
RR: relative risk
SF: synergy factor
TDT: transmission disequilibrium test
